# A System for the Real-Time Detection of the U-Shaped Steel Bar Straightness on a Production Line

**DOI:** 10.3390/s25133972

**Published:** 2025-06-26

**Authors:** Yen-Jen Chen, Yu-Hsiu Yeh, Jen-Fu Yang

**Affiliations:** 1 Department of Electronic Engineering, Ming Chi University of Technology, New Taipei City 243303, Taiwan; x599123@gmail.com (Y.-H.Y.); jfyang@mail.mcut.edu.tw (J.-F.Y.); 2 Center for Artificial Intelligence and Data Science, Ming Chi University of Technology, New Taipei City 243303, Taiwan

**Keywords:** steel inspection, straightness detection, AI object detection, image processing, automated optical inspection

## Abstract

This study develops an algorithm and a system for steel straightness detection, which combines object detection, edge detection, line detection, clustering, stitching, and bending recognition. The algorithm detects the contour of U-shaped steel bars with widths of 100 mm, named U100, or 150 mm, named U150, and lengths of 8, 10, 12 m. The algorithm uses object detection to extract the center point of the U-shaped bottom as a reference point and line detection to extract lines in the contour. The algorithm selects one-stage or two-stage edge detection based on the light source. Two-stage edge detection enhances the contour features when the light source is insufficient. After contour detection, some parts of the contour disappear due to the light source. The algorithm stitches all lines with an angle difference within ∆θ degrees into one straight line *L* based on the angle of the longest line. If the length of *L* exceeds the threshold value MLL, the steel bar is straight; otherwise, it is bent. ∆θ and MLL are used to set the acceptable bending degree. The experiment results show that the algorithm detects 123,128 steel bars in 193 h with an average accuracy of 99.64% for straight steel and an average recall of 95.70% for bent steel. The contribution of this study is the development of a real-time algorithm and its corresponding system for steel straightness determination in a steel factory, ensuring accurate and efficient assessment of steel quality in an industrial setting.

## 1. Introduction

The steel industry is a key sector of the global manufacturing industry, supplying steel materials for construction, manufacturing, and many other applications. To ensure the quality of steel bars, various countries have established strict quality standards, such as CNS 2473 [1], JIS G3101 [2], ASTM A36 [3], etc.

The current method for steel bar straightness inspection relies heavily on manual assessment, a process that is not only protracted and labor-intensive but also susceptible to discrepancies arising from subjective human judgment, thereby compromising consistency in quality control. The vast number of steel bars requiring inspection exacerbates the issue, as manual inspections are prone to errors and oversights due to inspector fatigue. These factors collectively have a detrimental influence on both production efficiency and the maintenance of stringent product quality standards. The precise determination of steel product straightness and curvature is crucial for ensuring structural integrity and optimal performance across critical sectors, including construction, automotive manufacturing, and energy production. Over the past decade, a lot of research literature has documented significant advancements in non-contact measurement methodologies, driven by the increasing demand for automation and enhanced precision in industrial environments.

Various techniques have been applied to measure straightness in a range of industrial settings. For instance, laser triangulation, as reported in [4], provides non-contact inspection with remarkable precision (up to 0.01 mm), although it is often associated with high costs and increased complexity. Alternatively, machine vision approaches, such as those utilizing OpenCV and Canny edge detection, as described in [5], offer a more cost-effective solution; however, these methods tend to be vulnerable to lighting fluctuations and generally deliver lower precision compared to laser-based systems. Moreover, a dual-sensor configuration introduced in [6] has been shown to improve the robustness of railway rail measurements, yet its applicability appears limited to that specific domain. Historically, laser position detectors (LPDs) have been employed to assess the straightness of drilling pipes [7], and edge detection algorithms have also been used to evaluate the curvature of hot-rolled steel bars [8]. The authors of [9] present an innovative defect detection system that utilizes best-fit polynomial interpolation to evaluate the condition of outer surfaces. Their approach enhances feature extraction capabilities, enabling the detection of multiple surface anomalies, including flatness deviations, waviness, blobs, and curvature faults. The authors of [10] present a laser-based method for straightness detection in steel products. This method is based on laser triangulation technology, integrated with robotic systems and connected to a software platform, achieving a precision of up to 0.01 mm. The authors of [11] present three primary methods for evaluating straightness: the maximum deviation method, the least squares method, and the minimum zone method. All three approaches use the two endpoints of a line as a reference and select multiple measurement points along the line. The distance between each measurement point and the reference line is calculated, and different formulas are applied depending on the method to determine the straightness. The authors of [12] present a hot-rolled steel edge straightness detection method based on the Hough transform. The algorithm consists of three main steps: (1) preprocessing, (2) Hough transform, and (3) straightness detection. Based on the linear equations obtained from the Hough transform, the distance from each pixel to the detected line is calculated. The square root of the sum of squared distances is then computed to derive a bending coefficient. If the coefficient exceeds 20%, the steel is considered bent.

Overall, the comparative analysis underscores the inherent trade-offs between precision, cost, and practical applicability, providing valuable insights into selecting the most appropriate measurement method for various industrial requirements.

### 1.1. Research Topic

To enhance the automation of steel bar straightness detection, an on-site study has been conducted at a steel factory known for high-volume, continuous production. The investigation addressed the challenges imposed by rigorous, standard-based quality inspections required to maintain minimal deviations. According to the production manager, each order yields thousands to tens of thousands of steel bars, with individual production lines manufacturing between 2 and 5 reinforcing bars per batch. Furthermore, the production process operates continuously, with cycle durations ranging from a few hours to up to two or three days.

**Figure 1:**
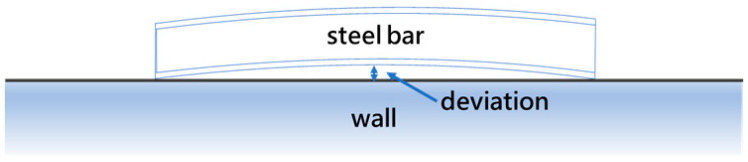
A steel bar bend deviation.

Steel bar quality inspections are conducted by shift workers in accordance with the CNS 1490 G1011 standard, with straightness being a critical parameter. For I- and T-beams, the acceptable straightness tolerance is 0.20% of the bar’s length, while for other sections it is 0.30%. For example, a 10-m U-shaped bar must exhibit a deviation of no more than 30 mm (see Figure 1).

**Figure 2:**
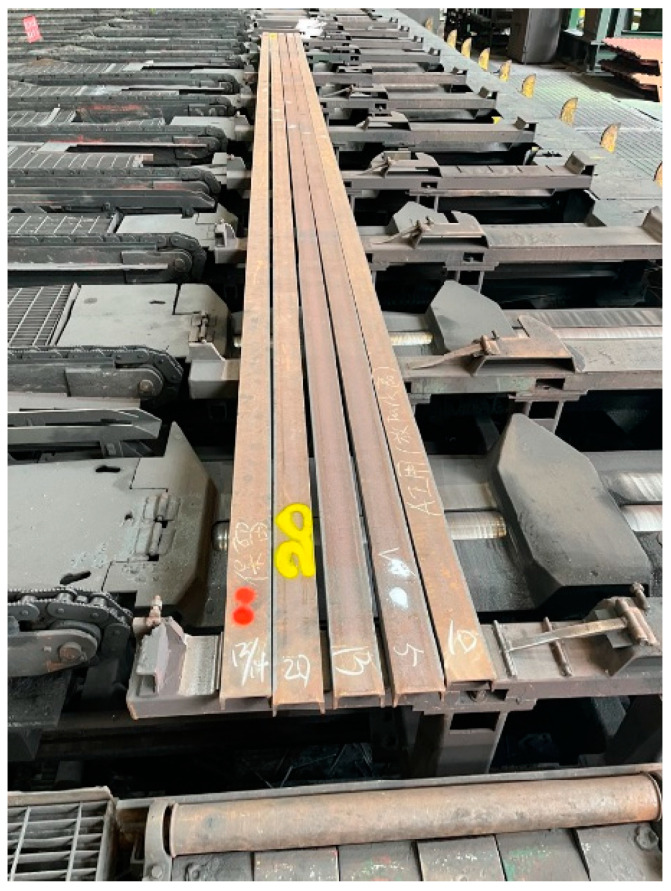
Sample steel bars used for establishing inspection standards. (There are some Chinese words on the steel bars such as “保留” and “AI用”, which mean “Reserved” and “Used for the AI field”, respectively).

For a 10-m-long steel bar, the acceptable amount of bending for U-shaped steel during factory production is 20 mm. If the bend reaches 20 mm or more, the steel is considered non-compliant. During inspection, operators visually evaluate steel bar straightness using footage captured by a fixed 1080P camera along the production line. The camera digitizes and transmits the video via a DVR to a 22-inch monitor for real-time assessment by on-site personnel, while the DVR concurrently compresses, stores, and streams the footage to a centralized control room. As illustrated in Figure 2, although the second and fourth U-shaped steel bars from the right appear bent, precise measurements indicate deviations of 10, 14, 13, 20, and 5 mm from left to right. The steel bar with the 20 mm deviation, indicated by the red arrow, is non-compliant.

In this study, the classification for quality control is based on test samples provided by the manufacturer. As shown in Figure 2, five steel bars, each 10 m in length, were tested. From left to right, they are labeled 13/14, 20, 13, 5, and 10. These values represent the deviation in bend (in millimeters), with “13/14” indicating a deviation between 13 mm and 14 mm.

Manual inspection of steel bars presents several challenges. Extended visual assessments often lead to operator fatigue, which can result in non-compliant bars being overlooked. Additionally, there is variability in inspection outcomes among different personnel, especially when measurements are close to the tolerance limits set by CNS 1490 G1011. The high-pressure production environment further increases the risk of visual inspection errors. To address these issues, this study proposes the development of a real-time steel bar straightness detection system, which will be evaluated using U-shaped steel bars with widths of 100 mm (U100) and 150 mm (U150).

### 1.2. Technical Bottlenecks and Limitations

Based on extensive on-site investigations, the automation of steel inspection faces several challenges. First, abnormal samples are extremely rare, with bent U-shaped steel bars accounting for less than 0.1% of total production, although during production, steel heat treatment or machining can cause bending, and even after passing through the straightener, some steel bars remain bent. Second, as illustrated in Figure 2, the steel bars move along the production line at a speed of 2–3 m per second, making conventional methods such as laser measurement or robotic arms impractical. Third, real-time processing is imperative: given that 10-m steel bars pass through the camera’s field of view in approximately 70 frames in 2.3 s, the system must complete the straightness error calculations for 2–5 bars within this time frame. Finally, the mixed illumination environment—comprising daylight, fixed lighting, and crane lights—creates localized bright spots that disrupt the accurate detection of steel bar contours.

To address these issues, this study proposes an AI-driven image processing solution to analyze camera footage in real time and detect bent steel bars. Detailed descriptions of the system architecture and algorithms are provided in Section 2.

## 2. System Architecture and Algorithm

This study introduces a novel algorithm and system for the automated recognition of steel bar straightness, designed to rapidly and accurately assess the conformance of U-shaped steel bars of varying dimensions to established straightness criteria. The methodological framework encompasses two primary components: a detailed exposition of the system architecture, presented in Section 2.1, and a comprehensive description of the algorithmic approach, delineated in Section 2.2.

### 2.1. System Architecture

**Figure 3:**
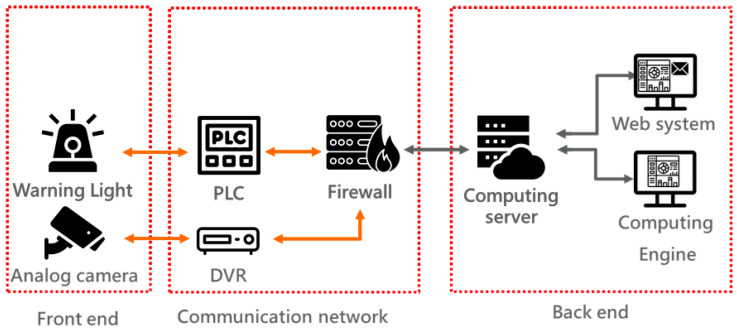
System architecture of the steel bar straightness recognition system.

The proposed steel bar straightness recognition system integrates a camera, a digital video recorder (DVR), a computational server, a programmable logic controller (PLC), and a visual warning indicator. The system architecture, as depicted in Figure 3, operates as follows: the camera captures real-time imagery of the steel bars and transmits these data to the DVR via a coaxial cable. Subsequently, the DVR streams the video feed to the computational server where a Computing Engine, in conjunction with the steel bar straightness recognition algorithm, processes the imagery to quantify steel bar straightness. If the measured straightness deviates from predefined standards, the server sends a signal to the PLC, which then activates the warning indicator to alert on-site personnel. Additionally, a web-based user interface enables real-time monitoring of steel bar production and recognition status by authorized personnel.

### 2.2. Algorithm

**Figure 4:**
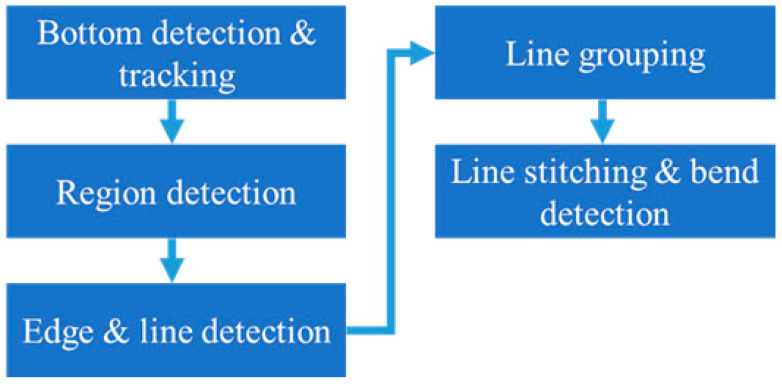
A flowchart of the steel bar straightness recognition algorithm.

The proposed algorithm employs a five-stage process to determine steel bar straightness: bottom detection and tracking, region detection, edge and line detection, line grouping, and line stitching and bend detection. The algorithmic workflow is illustrated in Figure 4. Each stage is detailed in the subsequent sections.

#### 2.2.1. Bottom Detection and Tracking

The steel bar production process is characterized by batch processing, with each batch containing one to five steel bars, as illustrated in Figure 2. Detection begins when the U-shaped bottom profile of the steel bars becomes visible as they fully enter the camera’s field of view.

**Figure 5:**
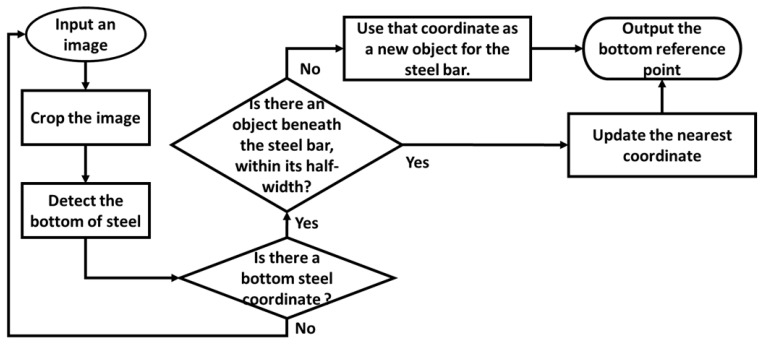
The bottom detection and tracking algorithm.

**Figure 6:**
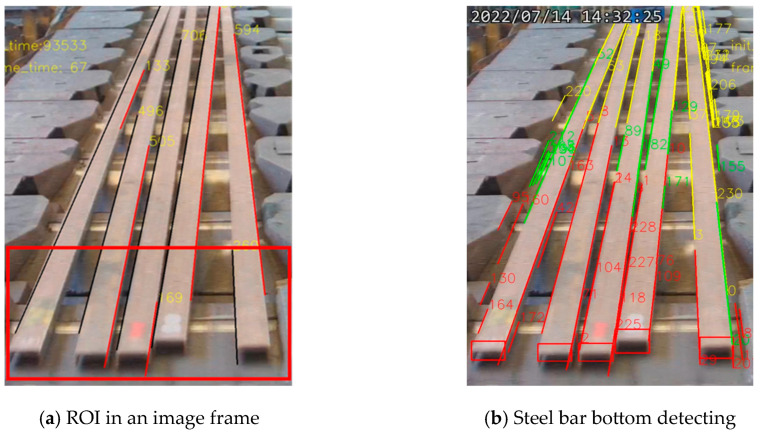
A region of interest for detecting the bottoms of steel bars.

The steel bar bottom detection and tracking algorithm, illustrated in Figure 5, begins by defining a region of interest (ROI) for bottom detection, as shown in Figure 6a. The input image is then cropped to isolate the bottom recognition region, facilitating the identification of the steel bar bottoms, as shown in Figure 6b with the red rectangle markers.

The detection of steel bar bottoms is accomplished using YOLOv5, which leverages its ability to identify the characteristic U-shaped morphology of the bottom region. This process generates an object descriptor, denoted as *fs*, encapsulating the class label, confidence score, and the coordinates defining the bounding box around the bottom region. The centroid, *fs*(*x*, *y*), of this bounding box is then computed from the lower-left and upper-right coordinates. Subsequently, a list, as expressed in Equation (1), is generated for each frame, comprising the centroids of all detected bottom regions.

(1) $${list}_{fs}=[{fs}_{1}\left(x_{1},y_{1}\right),{fs}_{2}\left(x_{2},y_{2}\right),\ldots ,{fs}_{n}\left(x_{n},y_{n}\right)]$$

After the initial detection of steel bar bottom objects, a continuous tracking methodology is employed. For each subsequent frame, the detection process is repeated, generating an updated list of bottom detection objects, denoted as ${list}_{fsnew}$. The preceding frame’s object list is correspondingly designated as ${list}_{fsori}$. Given the upward trajectory of the steel bars within the frame, as depicted in Figure 5, inter-frame horizontal displacement is utilized to ascertain object correspondence between ${list}_{fsnew}$ and ${list}_{fsori}$. This horizontal displacement, *md*, is mathematically defined by Equation (2).

(2) $$md=|{fs}_{m}(x)-{fs}_{n}(x)|$$

Let ${fs}_{m}(x)$ and ${fs}_{n}(x)$ represent the x-coordinates of objects within ${list}_{fsnew}$ and ${list}_{fsori}$, respectively. By comparing ${fs}_{m}(x)$ against elements in ${list}_{fsori}$, a set of displacement values, *md*, is computed. If any computed *md* is less than half the bounding box width, it confirms that ${fs}_{m}(x)$ corresponds to an object in the preceding frame. Conversely, if all computed *md* values exceed half the bounding box width, ${fs}_{m}(x)$ is identified as a new object. This new object is then appended to ${list}_{fsori}$, completing the object tracking process, as detailed in Equation (3).

(3) $$if\;md\leq \frac{{fs}_{width}}{2},\;{fs}_{m}={fs}_{n}$$

#### 2.2.2. Region Detection

The steel bar production process requires adjustments to meet diverse customer specifications, resulting in varying steel bar lengths. Consequently, the straightness detection threshold must dynamically adapt to these length variations. Using fixed parameters for anomaly detection would lead to inaccurate straightness assessments.

**Figure 7:**
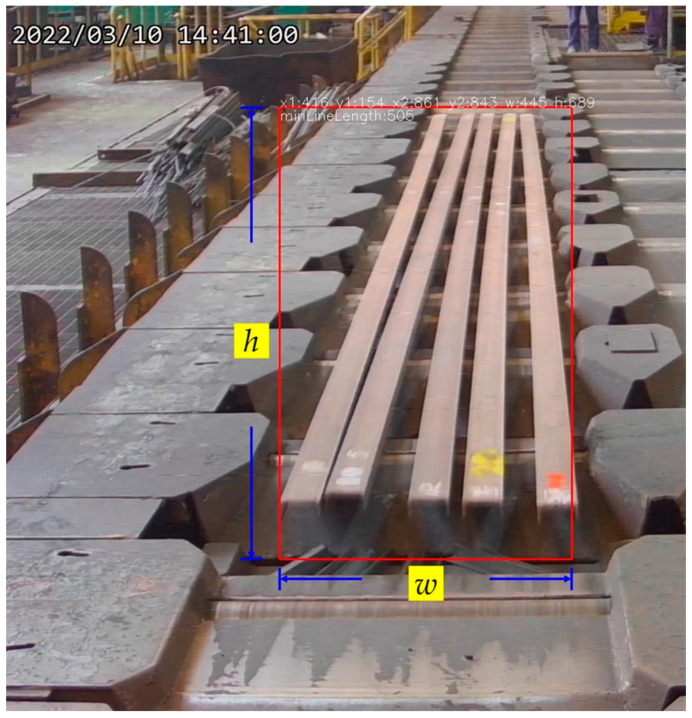
The bounding box for the entire batch of steel bars during an inspection.

Region detection aims to establish a dynamic bending detection standard for different steel bar models by using the bounding box length of the steel bar batch, obtained through object recognition, as a reference. A YOLOv5 model, trained with collected and labeled data using a methodology similar to the bottom detection process, is developed to recognize the bounding box encompassing the steel bar batch. Figure 7 presents the recognition results. The bounding box coordinates are denoted as ($x_{1}$, $y_{1}$) and ($x_{2}$, $y_{2}$), representing the lower-left and upper-right corners, respectively. The width and height of the bounding box are represented by *w* and *h*. The minimum line length (MLL) refers to the minimum acceptable length of the straight edge of a steel bar.

The process of defining the inspection standards for bent steel bars is illustrated in Figure 7. First, the five sample steel bars are placed on the production line and rearranged in a different order. The production line is then activated to transport the samples into the inspection area. This procedure is repeated to simulate actual production conditions.

According to the manufacturer, the purpose of quality control is to identify steel bars with bend deviations of 20 mm, 13/14 mm, and 13 mm. As shown in Figure 7, the steel bars from left to right have deviation values of 13 mm, 10 mm, 5 mm, 20 mm, and 13/14 mm. In this study, the parameter MLL (minimum line length) is used as the threshold for detecting bend deviations.

MLL is defined as the threshold value for the length of edge segments detected along the steel bar, measured in pixels. Through image processing, edge detection is performed on the steel bars, resulting in several line segments along the left and right edges. If these segments have an angle difference within ∆θ degrees based on the angle of the longest line segment, they are sequentially stitched to form the complete edge line of the steel bar. The parameter ∆θ is used as the threshold for the acceptance of stitching. ∆θ and MLL are used to set the acceptable bending degree. If the length of this edge line exceeds the MLL, the steel bar is considered straight; otherwise, it is classified as bent.

As shown in Figure 7, at a certain point during the transport process, the three steel bars on the right (with bend deviations of 13/14 mm, 20 mm, and 13 mm) completed edge detection within the inspection area. The lengths of both the left and right edge lines for each of these three bars are found to be less than 505 pixels. The height of the bounding box encompassing the entire batch of steel bars is 689 pixels. Based on this, a conversion factor between the bounding box height and the edge line length is calculated as 505/689 = 0.733.

This factor is defined as the steel bar bending detection coefficient. During production, if the height of the bounding box for a batch of steel bars is denoted as h, then the threshold for the edge line length, referred to as MLL (minimum line length), is calculated as Equation (4), where λ = 0.733. If both the left and right edge lines of a steel bar exceed this MLL value, the bar is considered straight; otherwise, it is classified as bent.

(4) h∗λ=MLL

λ is used to adjust the MLL based on varying *h*. For example, for steel bars of 8, 10, and 12 m in length, the conversion for ${MLL}_{k}$ and $h_{k}$ is illustrated in Equation (5), where k represents 8, 10, and 12.

(5) $$\lambda =\frac{{MLL}_{8}}{h_{8}}=\frac{{MLL}_{10}}{h_{10}}=\frac{{MLL}_{12}}{h_{12}}$$

#### 2.2.3. Edge and Line Detection

**Figure 8:**
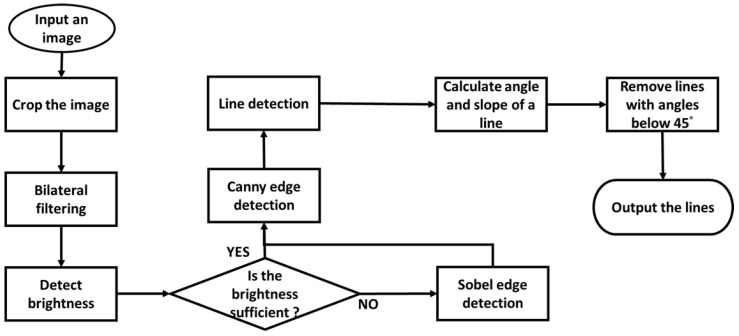
The edge and line detection flowchart.

The objective of edge and line detection is to accurately delineate the contours of steel bars within captured images. To achieve robust contour extraction, this paper proposes a methodology incorporating image enhancement techniques. The procedural workflow is illustrated in Figure 8.

Upon successful detection of steel bar bottom objects, the edge and line detection algorithms are initiated. Conversely, in the absence of detected bottom objects, these subsequent processing stages are bypassed. Prior to edge detection, image cropping and bilateral filtering are applied. Image cropping isolates a rectangular region of interest within the camera-captured image, thereby reducing computational overhead for subsequent image processing. Bilateral filtering serves to enhance image features.

**Figure 9:**
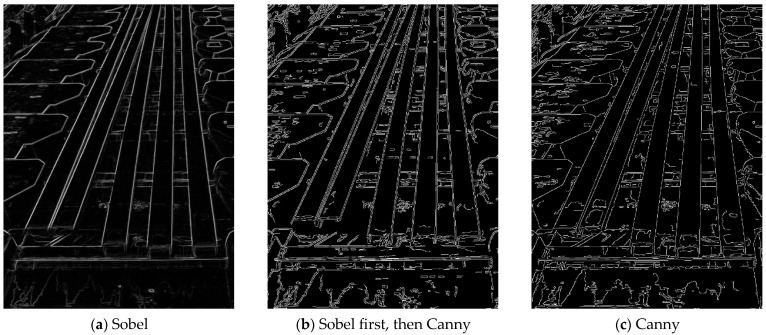
Edge detection for steel bars.

This study employs one-stage and two-stage edge detection to reconstruct the contour of steel bars. The choice between one-stage and two-stage methods is based on the threshold value of the average brightness, $V_{thr}$, which is set to 116 in this study. The first stage utilizes the Sobel operator with convolution to generate coarse edge maps, as illustrated in Figure 9a. Subsequently, the Canny edge detector refines these coarse edges, yielding precise edge contours, as shown in Figure 9b. Conversely, when the V value exceeds $V_{thr}$, a one-stage Canny edge detection method is applied, resulting in the direct generation of steel bar edges, as depicted in Figure 9c.

**Figure 10:**
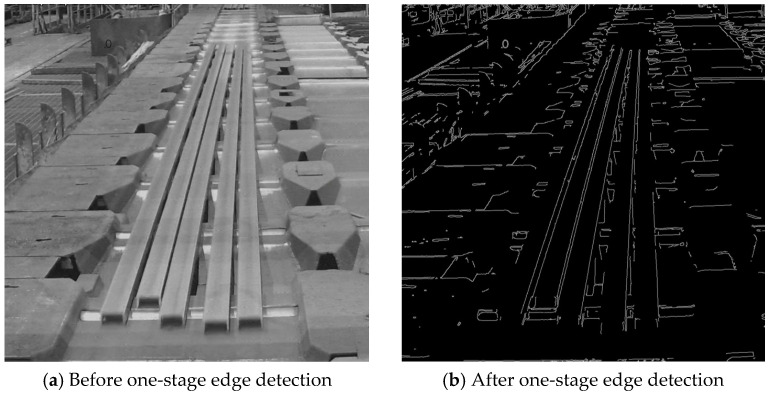
One-stage edge detection for steel bars.

One-stage edge detection: One-stage edge detection reconstructs the contour using the Canny edge detection algorithm. This method is applied when the average brightness of the input image exceeds the threshold value, $V_{thr}$. Brightness levels range from 0 to 255, where 0 represents the darkest and 255 the brightest intensity. As shown in Figure 10a, the calculated average brightness is 123.17, which is above the threshold. Therefore, one-stage edge detection is used, and the resulting image after processing is presented in Figure 10b.

**Figure 11:**
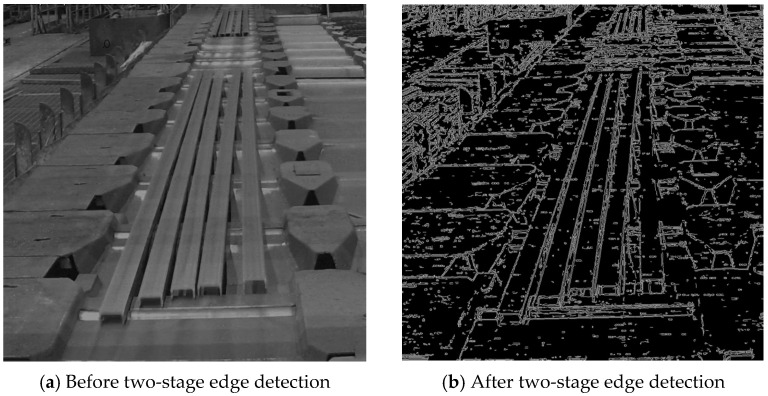
Two-stage edge detection for steel bars.

Two-stage edge detection: Two-stage edge detection reconstructs the contour by sequentially applying the Sobel and Canny edge detection algorithms. This method is used when the average brightness of the input image falls below the threshold value, $V_{thr}$. As shown in Figure 11a, the calculated average brightness is 82.35, which is below the threshold. In this case, the two-stage approach is applied, where Sobel edge detection is followed by Canny edge detection to reconstruct the contour. The final processed result is presented in Figure 11b.

The goal of line detection is to reconstruct the steel bar’s contour based on its geometric characteristics. The Hough transform algorithm is utilized for straight line detection, as implemented in OpenCV. A key parameter in this process is maxLineGap, which controls the merging of collinear and adjacent line segments. Higher values of maxLineGap promote more extensive line merging, while lower values do the opposite. To reduce noise, line segments shorter than 50 pixels are filtered out.

Before detection, a brightness threshold $V_{thr}$ is determined based on the median intensity within the detection region. If the median brightness exceeds $V_{thr}$, a maxLineGap value of 20 is applied; otherwise, a value of 50 is used to accommodate lower-contrast conditions.

**Figure 12:**
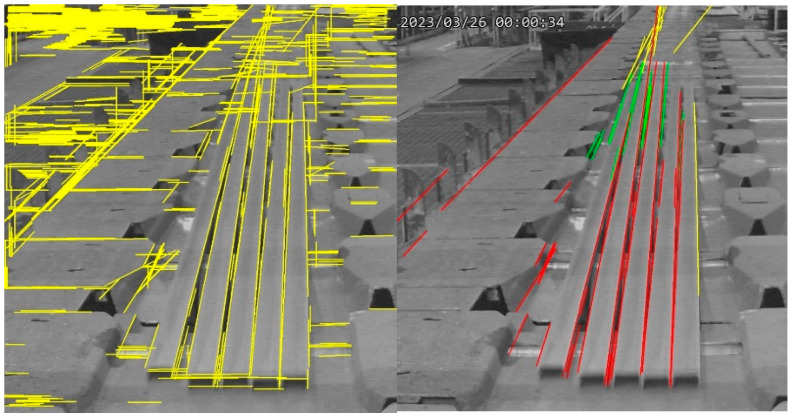
Noise filtering after edge detection.

Post-detection, noise reduction is performed by eliminating all lines with slopes within ±45 degrees of the horizontal axis, effectively removing near-horizontal lines. This noise filtering procedure is illustrated in Figure 12.

#### 2.2.4. Line Grouping

The purpose of line grouping is to associate detected line segments with their corresponding steel bars, based on the line detection output. Following line detection, a set of line segments is obtained, each defined by the coordinates of its start and end points.

A line grouping algorithm is employed to calculate the slope and angle of each line segment, extending these segments to the bounding box (bbox) of the steel bar’s bottom object detection. Subsequently, the difference between the line segment’s y-coordinate ($y_{houghline}$) and the bounding box’s y-coordinate ($y_{bbox}$) is computed. Using the line segment’s slope ($m_{hough}$), the x-coordinate ($x_{line}$) of the line segment at the bounding box is then derived, as shown in Equation (6).

(6) $$x_{line}=x_{hough}-\frac{y_{bbox}-y_{houghline}}{m_{hough}}$$

**Figure 13:**
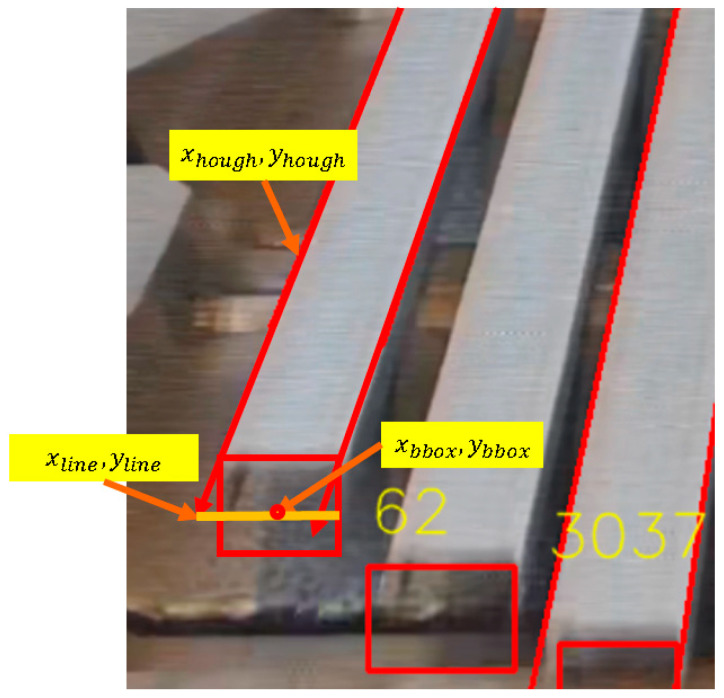
Line grouping for steel bars.

Line segments, defined by their slope, angle, and (*x*, *y*) coordinates, are matched to the bounding boxes with the nearest x-coordinates, thereby assigning each line segment to its corresponding steel bar. This association indicates that the line segments belong to the steel bars enclosed within the bounding boxes, as illustrated in Figure 13.

After assigning line segments to their respective bounding boxes, the distance (d) between each line segment and its corresponding bounding box is calculated, as defined by Equation (7).

(7) $$d=x_{bbox}-x_{line}$$

The lateral position of each line segment relative to its bounding box is then determined. If d < 0, the line segment is classified as being on the left side of the bounding box; otherwise, it is classified as being on the right side, as expressed in Equations (8) and (9).

(8) $$if\;d<0,line\in {linegroup}_{left}$$

(9) $$if\;d>0,line\in {linegroup}_{right}$$

#### 2.2.5. Line Stitching and Bend Detection

The objective of line stitching is to mitigate gaps between line segments detected on the steel bar, thereby facilitating accurate straightness assessment. Line segments are stitched based on angular proximity, reducing the impact of gaps on straightness measurement. According to national production standards and manufacturer specifications, a straight steel bar exhibiting a 5 mm bow-shaped bend manifests an angular difference of 0.3 degrees between its extremities. Incorporating a 10% margin of error yields a threshold of 0.33 degrees.

The line stitching algorithm calculates line segment angles for each bounding box of the steel bar bottom. For each line group, the angle of the longest line segment is designated as the reference angle, $\theta_{b}$. Subsequent line segment angles are compared against $\theta_{b}$, and if the ∆θ is less than 0.33 degrees, the line segment is merged with the reference line, effectively treating them as a single entity, as defined by Equations (10) and (11).

(10) $$∆\theta =\theta_{b}-\theta_{line}$$

(11) $$if\;\left|∆\theta \right|<0.33,\;Line\;splicing$$

Following line stitching, line segments are adjusted by multiplying the stitched line length, $L_{s}$, by $\sin\theta_{b}$, yielding $L_{m}$, and aligned to a 90-degree reference position for subsequent straightness comparison, as specified in Equation (12).

(12) $$L_{m}=L_{s}*\sin\theta_{b}$$

For bend detection, the minimum line length parameter MLL, as detailed in Section 2.2.2, is employed to evaluate adherence to straightness criteria. The lengths of each side of the steel bar, $L_{mr}$ and $L_{ml}$, are compared against the MLL. If both sides align, the steel bar is deemed straight.

To satisfy factory requirements, a more stringent dynamic threshold coefficient, λ, is applied during the initial 1.5 h of production, implementing a rigorous standard for early-stage detection. This ‘strict mode’ aims to enhance detection rates and improve production yield.

In specific scenarios, bending detection may persistently classify certain steel bars as bent, particularly those located at the track’s edges or when the camera operates in grayscale mode. This is attributed to the edge detection algorithm’s inability to clearly delineate edges in these instances, resulting in insufficient line segments for stitching. To address this, the criteria are relaxed for edge steel bars and grayscale images, permitting a single line segment satisfying the MLL to qualify as straight. The general and relaxed modes for straightness determination are defined by Equations (13) and (14), respectively.

The system uses a rule-based adaptability mechanism to handle varying lighting conditions. It continuously monitors image brightness and switches to relaxed mode under low luminance. To address high-glare conditions, adjustments were made during the camera installation phase. By optimizing the camera’s position, angle, and configuration settings, glare was effectively minimized.

(13) $$L_{mr}>MLL\;and\;L_{ml}>MLL$$

(14) $$L_{mr}>MLL\;or\;L_{ml}>MLL$$

where $L_{mr}$ denotes the stitched and transformed line representing the comparison between the steel bar’s right edge and the MLL, and $L_{ml}$ denotes the corresponding line for the left edge.

**Figure 14:**
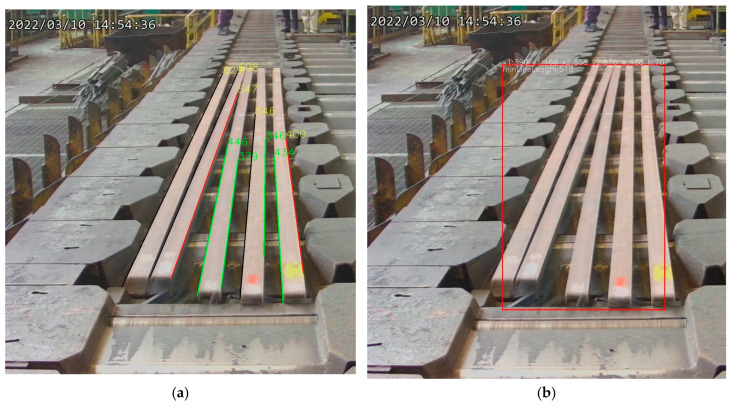
Bend detection of the steel bars. (**a**) Left and right edge lines of steel bars, (**b**) Bounding box of the batch of steel bars (Height = 707 Pixels).

As shown in Figure 14b, with a vertical height *h* = 707, the MLL is is calculated using Equation (4) as 518 (518 = 707 × 0.733). In practice, the edge line lengths must be corrected based on the steel bar’s tilt angle, denoted as $\theta_{b}$. The corrected length is obtained by multiplying the original length by *sin*$\theta_{b}$, as defined in Equation (10).

Referring to Figure 14a, the steel bars from left to right have curvature deviations of 10, 5, 13, 13/14, and 20 mm. Due to lighting conditions, the algorithm allows the leftmost and rightmost bars to be considered straight if either edge line exceeds the MLL, as specified in Equation (14). For the middle bars, both edge lines must exceed the MLL to qualify as straight, according to Equation (13).

In Figure 14a: The 10 mm bar has a left edge line of 629 pixels with a tilt angle of 64.54°. According to the calculation in Equation (12), the corrected length is 568 (629 × sin64.54° = 568). Since length > MLL (568 > 518), it is classified as straight.

The 5 mm bar has a left edge line of 608 pixels at 66.78°. The corrected length is 559 (608 × sin66.78° = 559). The right edge line of 547 pixels at 71.60°, corrected to 519 (547 × sin71.60° = 519). Both exceed the MLL, so this bar is also straight.

For the 13/14 mm bar, the left edge line is 546 pixels at 85.04°, corrected to 544 (546 × sin85.04° = 544), which exceeds the MLL, but the right edge line is only 346 pixels and does not meet the threshold. Therefore, this bar is bent. The 13 mm and 20 mm bars have edge lines that do not exceed the MLL on either side and are thus classified as bent.

## 3. Implementation and Experimental Results

This section describes the implementation environment, presents the experimental results, and analyzes the outcomes.

### 3.1. Implementation Environment

The implementation environment consists of two servers, with hardware specifications detailed in Table 1. This dual-server setup ensures high system availability, allowing for seamless failover in the event of a workstation malfunction.

The deployment context is a steel bar production line within a manufacturing facility. An analog camera captures imagery of the steel bar production process and transmits the data to a digital video recorder (DVR). The DVR converts the analog signals into a digital format, compressing and storing them on a hard disk. Using the real-time streaming protocol (RTSP) streaming service provided by the DVR, the server establishes a connection and employs the ffmpeg decoder to relay camera imagery to the computer for real-time recognition.

### 3.2. Implemented System and Experimental Results

**Figure 15:**
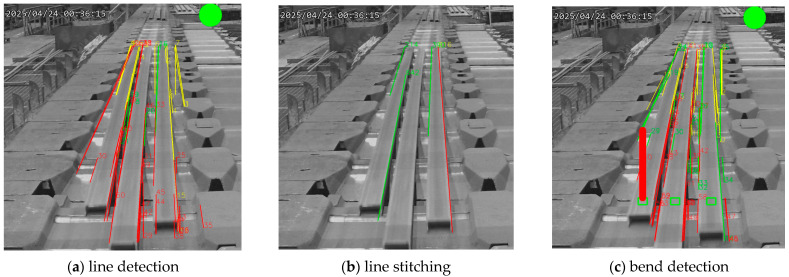
Implementation results of real-time straightness detection (as shown in (**a**,**c**), where the green circles indicate the system is in general mode but not relaxed mode; however, (**b**) is generated in debug mode, without green circle, to show the line stitching between (**a**,**c**)).

The steel bar straightness recognition process involves the following steps: Initially, the Hough transform method is applied to detect and visualize straight lines within the edge-detected image, assigning distinct colors to the respective line coordinates. This step includes straight line detection as illustrated in Figure 15a. Subsequently, the stitched straight segments are grouped, yielding the final stitched line results, as shown in Figure 15b. Finally, in case of anomaly detection, a red indicator is displayed at the bottom of the steel bar, as shown in Figure 15c.

**Figure 16:**
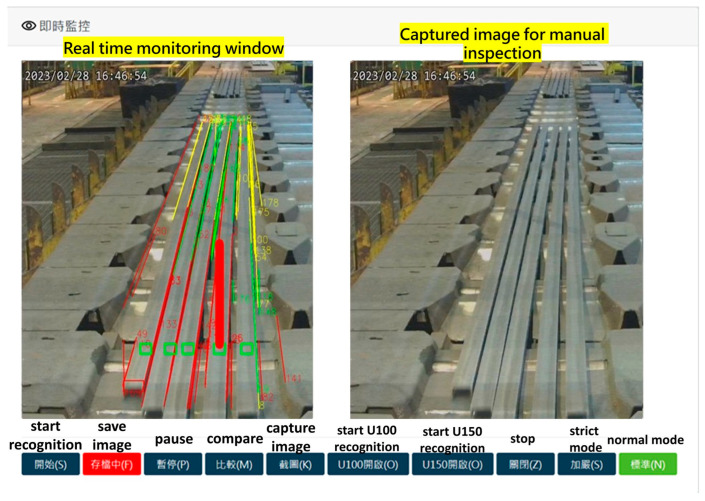
A web-based user interface of the real-time straightness recognition system. (The names of the buttons are in Chinese; their corresponding names in English are shown above the buttons).

The web-based user interface provides real-time video monitoring, allowing operators to visualize the steel bar recognition process. Operators can dynamically adjust recognition parameters to meet current production specifications. For verification purposes, the video feed can be paused, enabling a comparative analysis between the displayed image and captured frames, as illustrated in Figure 16.

This study evaluates a machine vision-based system designed to identify bent steel bars (considered defects) during live testing conducted on-site between October 2022 and October 2023. The dataset, collected over 193 h, includes 123,128 steel bars, categorized by types (U100, U150). Based on the defined parameters ∆θ and MLL, the classification results of the detection algorithm are defined as follows:

- True positive (TP): A correctly identified bent steel bar. This means that after reconstructing and stitching its contour segments using the angular tolerance ∆θ, the total length of the resulting straight segments is less than the MLL.
- False positive (FP): A straight steel bar incorrectly classified as bent. In this case, the reconstructed straight segment length using ∆θ is less than the MLL, even though the bar is actually straight.
- True negative (TN): A correctly identified straight steel bar. Its contour, when reconstructed and stitched using ∆θ, results in a total straight segment length greater than the MLL.
- False negative (FN): A bent steel bar incorrectly classified as straight. Although the bar is actually bent, the reconstructed straight segment length using ∆θ exceeds the MLL, leading to a misclassification.

This analysis examines the system’s accuracy, recall, and reliability, offering insights into its practical applicability in industrial environments. Accuracy and recall metrics were calculated using Equations (15) and (16), respectively. The detection outcomes were classified as follows:

(15) $$Accuracy=\frac{TP+TN}{TP+FP+TN+FN}$$

(16) $$Recall=\frac{TP}{TP+FN}$$

The dataset was gathered from nine live test periods conducted on-site, inspecting a total of 123,128 steel bars over 193 h. The steel bars are classified into U100 and U150 types, with varying quantities across each test period. The overall classification outcomes are: 89 true positives (TP), 437 false positives (FP), 122,598 true negatives (TN), and 4 false negatives (FN). The system achieves an overall accuracy of 99.64% and a recall of 95.70%. Each test period’s duration, number of steel bars, and performance metrics are detailed in Table 2.

## 4. Discussion

This section provides a comprehensive analysis of the system’s performance in detecting bent steel bars. It begins by examining the experimental results, breaking down key findings and metrics. Next, it compares these results with previous research, highlighting advancements or differences. The section then discusses the efficiency of the algorithm by analyzing its time complexity. Finally, it concludes by discussing the cost-effectiveness of using this automated inspection system in a real-world manufacturing setting.

### 4.1. Analysis of Experiment Results

The system demonstrates consistently high accuracy across all test periods, ranging from 98.93% to 99.96%, with an overall accuracy of 99.64%. This indicates that the system correctly classifies the vast majority of steel bars, whether bent or straight. The high True Negative (TN) values (122,598 out of 123,128 total bars) reflect the system’s strong ability to identify straight steel bars, which dominate the dataset—a common scenario in manufacturing where defects are rare.

The recall, which measures the system’s ability to correctly identify bent steel bars (TP/(TP + FN)), ranges from 85.71% to 100%, with an overall recall of 95.70%. The recall is notably high in most test periods, achieving 100% in six out of nine tests, indicating that the system rarely misses bent steel bars (FN = 4 across all test periods). However, the system’s performance is slightly impacted by a relatively high number of false positives (FP = 437). False positives (FP) occur when straight steel bars are incorrectly classified as bent, potentially leading to unnecessary rejections.

The false negative (FN) count, representing missed bent steel bars, is notably low (FN = 4), occurring in only two test periods. For instance, during the test period from 2022/10/07 14:30 to 2022/10/08 10:30, 2 FN were recorded out of 19,053 U100 bars, resulting in a recall of 94.29%. Similarly, the test period from 2023/05/31 14:00 to 2023/06/01 10:00 had 2 FN out of 23,522 U100 bars, with a recall of 85.71%. These rare misses indicate the system’s high reliability in detecting bent steel bars. All steel bars classified as false negatives (FN) exhibited bending at the leading edge. In these cases, the bend occurred so close to the front end that the algorithm’s straight-line fitting still met the criteria for straightness, resulting in a misclassification. In such cases, the bent bar can be incorrectly classified as straight. However, the factory’s production supervisor has indicated that this type of end bend is exceptionally uncommon in their operations, as bending typically occurs in the middle section of the bar. This rare occurrence aligns with the low incidence of false negatives (FN) observed only in 4 out of 123,128 steel bars during a one-year real-world testing period.

False positives (FP), where straight steel bars are incorrectly identified as bent, arise from two primary environmental and imaging challenges. First, strong interfering light in the environment can create shadows at the base of the steel bar, resembling the U-shaped feature of a bent bar. This causes the system to misinterpret the shadow as the bar’s base, prematurely terminating the identification process and classifying the bar as bent. For instance, in the test period from 2023/06/14 14:00 to 2023/06/16 10:00, the high FP count (100) was influenced by such lighting conditions, as this test period spanned 44 h with potentially varying light exposure.

Second, camera jitter can cause blurred edge imaging of the steel bar. Even after image processing, the algorithm struggles to identify short straight-line segments for stitching. As a result, the stitched line may not be long enough to meet the straightness criterion, leading to a bent classification. This issue has contributed to the high FP counts in test periods, such as from 08/24 23:00 to 08/25 14:00 (15 h, 48 FP), where vibrations make the camera jitter.

### 4.2. Comparison with Previous Studies

Various studies have explored different approaches to measuring the straightness of steel, each with distinct advantages. Table 3 presents a comparative overview of various measurement systems employed in industrial contexts, focusing on precision, sample size, and application scenarios.

González et al. [5] present a non-contact measurement system for edge waviness in rolling mill steel sheets, operating on sheet lengths ranging from 25 to 35 m and at production speeds of up to 5 m/sec. Their system utilizes three synchronized monochrome CCD cameras with an RGB frame grabber and Canny edge detection, achieving error detection within a range of 25 to 150 mm/m. In contrast, our research employs a single-color camera with grayscale conversion to achieve similar computational efficiency. Both approaches incorporate image cropping, Canny edge detection, and Hough line-based contour reconstruction for anomaly detection, adhering, respectively, to ISO 9444 Part 2 (González et al.) and a factory standard of 2 mm/m (our work). In terms of real-time performance, the system presented in [5] is capable of processing images at a rate of 5 m/sec. In our study, the performance is evaluated by using video footage of 10-m-long steel bars passing through the production line. It takes 8.54 s for a single 10-m steel bar to fully present into the image frame. Based on this, our system is capable of processing approximately 1.17 per second.

Fang et al. [7] address the challenge of online straightness measurement in long seamless steel pipes, where automated solutions are scarce, by proposing a multi-tunnel laser position detector (LPD) system. This method achieves high precision, with a measurement precision better than 0.02 mm/m, and is designed for single-item batch production, specifically targeting drilling pipes. Our research introduces a detection methodology tailored for the batch production of one to five U-shaped steel bars.

Golkar et al. [9] present a real-time defect detection system for outer surfaces, employing best-fit polynomial interpolation to identify flatness, waviness, blobs, and curvature faults, validated on pipes and ceramic tiles with successful detection of various physical anomalies. While both Golkar et al.’s and our research utilize camera-based systems and build upon Canny edge detection for defect analysis, their work focuses on detecting minute surface defects on tiles, such as flatness deviations and curvature variations to assess straightness. In contrast, our research specifically concentrates on evaluating the straightness of steel using Hough line clustering and stitching techniques.

Valentina et al. [10] present a laser-based measurement method validated through simulation. Chang’an, H. et al. [11] also employ a laser-based measurement method, but their validation is conducted in a laboratory and has not yet been deployed on a production line. Bai, J. et al. [12] introduce a method to detect bending through changes in slope, and then classify the bending into five categories based on different bending degrees.

A summary of the studies, with actual tests or specified precision, is shown in Table 3. González et al. [5], Golkar, E. et al. [9], and the proposed scheme all utilize video processing techniques to provide real-time straightness detection. In contrast, Fang, S. et al. [7] employ a laser scanning approach. In terms of system cost, image-based systems are generally more economical than laser-based systems. While González et al. [5] demonstrate lower accuracy, their system achieves the highest processing speed among all, at a production speed of 5 m/s. Both Golkar, E. et al. [9] and the proposed scheme achieve comparable accuracy; however, the proposed scheme is validated with a large number of test samples and is currently in continuous operation on-site. It is capable of processing 1 to 5 steel bars every 0.5 s, demonstrating its superior performance.

The related studies and research on the straightness detection of steel bars are limited. Among the few, two notable studies include those by M. Li [6] and Bai, J. et al. [12]. M. Li [6] measured the straightness of rails using a dual-sensor setup; however, the experiment was conducted in a laboratory environment and was not applied to a production line. Bai, J. et al. [12] attempt to divide the steel bar into upper and lower halves and separately calculate the bending coefficient for each half. The bending coefficient is determined by identifying two line segments along the edge of the steel bar, with slopes denoted as K_1_ and K_2_. Then, based on the two endpoints that are farthest apart between these segments, the slope K of the connecting line is calculated. The bending coefficient, denoted as ξ (which is denoted as λ in the paper of Bai, J. et al. [12]), is obtained by averaging the absolute percentage differences between K_1_ and K, and K_2_ and K. A larger ξ indicates a greater degree of bending. Figure 5 in Bai, J. et al. [12] presents a sample in which the upper half has slopes K_1_ = −0.1759, K_2_ = −0.2323, and K = −0.2034. The corresponding bending coefficient, ξ, is calculated to be 13.86%. The advantage of this method lies in its ability to accurately capture the bending characteristics of both the upper and lower halves of the steel bar. Since precise calculation of the slope values (K) is required, high-quality imaging is essential—specifically, images in which the edges of the steel bars are sharp and clearly visible. In fact, the steel bar images presented in [12], such as those in Figures 2, 3, and 5 of [12], all exhibit clearly defined and sharp edges. However, the site addressed in this study is an environment where lighting conditions change over time due to both natural variation and the movement of internal machinery (such as overhead cranes). Under such conditions, the captured images often show fragmented and discontinuous edges of the steel bars. In scenarios where 3 to 5 steel bars need to be identified simultaneously, if the upper half of a steel bar is bent and its upper edge is detected as a series of discontinuous segments, it becomes difficult to correctly group these segments. As a result, it is not possible to determine which segments belong to which steel bar. Therefore, an effective approach is to iteratively connect short edge segments from the bottom of the steel bar upward, continuing the stitching process until no further connections can be made. The length of the longest resulting straight line serves as the optimal metric for evaluating the straightness of the steel bar—the longer the line, the straighter the bar. By setting a length threshold, steel bars with a certain degree of bending can be reliably detected. The study of [12] provides three sample cases to validate the correctness of its method. However, the proposed scheme of this paper is conducted in an environment with varying lighting conditions, which poses the challenge of not being able to detect bent steel bars with 100% certainty. Additionally, there is a risk of misclassifying straight bars as bent. To address these lighting variations, adaptive mechanisms were incorporated in the proposed scheme. Through testing of hundreds of thousands of steel bar samples, the method achieved an average recall rate of over 95%, and an accuracy rate exceeding 99%. These results highlight the novelty and superiority of this study. The novelty of the proposed scheme lies in its ability to perform real-time measurements across multiple manufacturing samples and to automatically adjust detection thresholds based on the length of the steel bars. This adaptive mechanism is designed to meet the dynamic requirements of production environments. In terms of superiority, the proposed system achieves higher precision than other video-based methods such as those provided by González et al. [5] and Golkar et al. [9]. Compared to laser-based techniques like Fang et al. [7], the proposed scheme offers a more cost-effective and scalable solution for high-volume production, without requiring modifications to the existing manufacturing setup.

### 4.3. Time Complexity of the Algorithm

The system proposed in this study is designed to support real-time processing, with its core functionality centered on the grouping and stitching of straight lines to reconstruct the contour of steel bars. The algorithm is analyzed as follows:

- Line grouping: Using the clustering algorithm described in Section 2.2.4, each line segment is processed once to determine its corresponding group. This procedure is executed *n* times in total, resulting in a time complexity of *O(n)*.
- Line stitching: After grouping, all line segments within the same group are assumed to belong to the contour of the same steel bar. Therefore, stitching is performed *n* times to merge line segments whose angular difference from the reference line is less than 0.33 degrees. This step also has a time complexity of *O(n)*.

In summary, the proposed algorithm achieves a linear time complexity of *O(n)* for the core processes of line grouping and stitching.

### 4.4. Cost-Effective Automated Inspection

The implementation of the described steel bar detection system represents a significant shift from a traditional manual visual inspection to full automation. This transition not only eliminates the inherent subjectivity and potential for human error associated with manual processes but also dramatically improves efficiency. The system reduces manual inspection by over 99%, moving from inspecting 123,128 bars manually to just 526 (TP and FP steel bars) using the automated system. Notably, the automated system demonstrates the capability to achieve comprehensive quality control by inspecting this significantly reduced sample size. Furthermore, the deployment of this automated system presents a highly cost-effective solution, with an estimated implementation cost of less than the unit price of a server (under USD 2000). This combination of enhanced accuracy, reduced inspection workload, and minimal investment underscores the transformative potential of this automated approach for steel bar quality control.

## 5. Conclusions

The steel bar straightness detection system presented in this study effectively mitigates the adverse effects of variable lighting conditions, including contour blurring under diverse illumination scenarios, contour discontinuity due to localized light sources, and detection challenges arising from insufficient luminance. Furthermore, the system incorporates a ‘strict mode’ adaptable to specific production requirements. Key findings include:

- Adaptive edge detection: Employing one-stage or two-stage methods based on varying lighting conditions to accurately capture the steel contour.
- Contour reconstruction: Using object detection to extract a reference point at the U-shaped bottom and line detection to reconstruct the contour by stitching segments with angular differences within a threshold (∆θ).
- Straightness detection: Classifying steel as straight if the reconstructed contour length (*L*) exceeds a defined threshold (MLL), with ∆θ and MLL setting the acceptable bending tolerances.

The experimental results show that over 193 h of production, the system processed 123,128 steel bars, achieving a 99.64% accuracy and a 96% recall rate for bent steel, with each batch detection completed within 0.5 s. After implementing the automation process, manual inspection work can be reduced by over 99%, decreasing from the original manual inspection of 123,128 steel bars to just 526 abnormal steel bars. Furthermore, the deployment of this automated system offers a highly cost-effective solution, with an estimated implementation cost lower than the unit price of a server.

However, certain limitations persist, particularly the potential for undetected bending at the leading edge of steel bars and compromised detection performance under grayscale imaging conditions. The primary contribution of this research lies in the development of an automated optical inspection (AOI) algorithm suite specifically designed for steel bar straightness determination in environments where light source manipulation is infeasible. This system enables accurate identification of straight steel bars and efficient segregation of bent steel bars, significantly improving inspection efficiency while maintaining cost-effectiveness.

In future research, to address the current limitation of the algorithm in detecting bends at the top end of steel bars, a complete contour of the steel bar will first be constructed. The bend detection mechanism will then be enhanced by analyzing angular variations along the full contour line.

## Figures and Tables

**Table 1:** Specifications of the straightness recognition system server.

Component	Specification
CPU	Intel I5-10400
Motherboard	Gigabyte B580M
Memory	Kingston 8GB DDR4 2666 *2 16G
SSD	ADATA SU800 512G
HDD	Seagate ST4000 4T
GPU	MSI GeForce RTX 2060 VENTUS 6G OC
Power Supply	550 W

**Table 2:** Experimental results of straightness detection for U100 and U150 productions.

Test Date and Time	Duration (Hours)	Number of Steel Bars	Types of Steel	TP	FP	TN	FN	Accuracy (%)	Recall (%)
2022 10/07 14:30 ~ 10/08 10:30	20	19,053	U100	33	53	18,965	2	99.71	94.29
2022 12/07 14:00 ~ 12/07 23:00	9	3281	U150	7	35	3239	0	98.93	100
2023 05/31 14:00 ~ 06/01 10:00	20	23,522	U100	12	90	23,418	2	99.61	85.71
2023 06/14 14:00 ~ 06/16 10:00	44	11,183	U150	10	100	11,073	0	99.11	100
2023 06/21 14:00 ~ 06/22 10:00	20	23,929	U100	10	85	23,834	0	99.65	100
2023 08/23 22:00 ~ 08/24 16:30	18.5	6409	U150	1	3	6405	0	99.95	100
2023 08/24 23:00 ~ 08/25 14:00	15	6876	U150	2	48	6826	0	99.30	100
2023 09/21 22:00 ~ 09/23 11:00	37	13,435	U150	1	6	13,428	0	99.96	100
2023 10/12 08:00 ~ 10/12 17:30	9.5	15,440	U100	13	17	15,410	0	99.89	100
Total/Average	193	123,128		89	437	122,598	4	99.64	95.70

**Table 3:** Comparison of various steel straightness measurement techniques.

Author	System	Precision	Samples	Measurement Times	Application
González et al. [5]	video processing (Hough line)	25–150 mm/m	3	Not Available	hot-rolled steel sheet manufacturing
Fang, S. et al. [7]	laser scanning	0.02 mm/m	1	Not Available	drilling pipe manufacturing
Golkar, E. et al. [9]	video processing (curvature)	3.25 mm/m	200	Not Available	ceramic tile mass production
Proposed scheme	video processing (Hough line)	2 mm/m	123,128	0.5 s for 5 steels	mass production of structural steel

## Data Availability

Data are unavailable due to privacy.
